# *FAM64A*: A Novel Oncogenic Target of Lung Adenocarcinoma Regulated by Both Strands of *miR-99a* (*miR-99a-5p* and *miR-99a-3p*)

**DOI:** 10.3390/cells9092083

**Published:** 2020-09-11

**Authors:** Keiko Mizuno, Kengo Tanigawa, Nijiro Nohata, Shunsuke Misono, Reona Okada, Shunichi Asai, Shogo Moriya, Takayuki Suetsugu, Hiromasa Inoue, Naohiko Seki

**Affiliations:** 1 Department of Pulmonary Medicine, Graduate School of Medical and Dental Sciences, Kagoshima University, Kagoshima 890-8544, Japan; keim@m.kufm.kagoshima-u.ac.jp (K.M.); k8802984@kadai.jp (K.T.); k8574402@kadai.jp (S.M.); taka3741@m2.kufm.kagoshima-u.ac.jp (T.S.); inoue-pulm@umin.net (H.I.); 2 MSD K.K., Tokyo 102-8667, Japan; nijiro.nohata@merck.com; 3 Department of Functional Genomics, Chiba University Graduate School of Medicine, Chiba 260-8670, Japan; reonaokada@chiba-u.jp (R.O.); shun72317@gmail.com (S.A.); 4 Department of Biochemistry and Genetics, Chiba University Graduate School of Medicine, Chiba 260-8670, Japan; moriya.shogo@chiba-u.jp

**Keywords:** lung adenocarcinoma, microRNA, *miR-99a-5p*, *miR-99a-3p*, *FAM64A*, tumor suppressor

## Abstract

Lung adenocarcinoma (LUAD) is the most aggressive cancer and the prognosis of these patients is unfavorable. We revealed that the expression levels of both strands of *miR-99a* (*miR-99a-5p* and *miR-99a-3p*) were significantly suppressed in several cancer tissues. Analyses of large The Cancer Genome Atlas (TCGA) datasets showed that reduced *miR-99a-5p* or *miR-99a-3p* expression is associated with worse prognoses in LUAD patients (disease-free survival (DFS): *p* = 0.1264 and 0.0316; overall survival (OS): *p* = 0.0176 and 0.0756, respectively). Ectopic expression of these miRNAs attenuated LUAD cell proliferation, suggesting their tumor-suppressive roles. Our in silico analysis revealed 23 putative target genes of pre-*miR-99a* in LUAD cells. Among these targets, high expressions of 19 genes were associated with worse prognoses in LUAD patients (OS: *p* < 0.05). Notably, *FAM64A* was regulated by both *miR-99a-5p* and *miR-99a-3p* in LUAD cells, and its aberrant expression was significantly associated with poor prognosis in LUAD patients (OS: *p* = 0.0175; DFS: *p* = 0.0276). *FAM64A* knockdown using siRNAs suggested that elevated *FAM64A* expression contributes to cancer progression. Aberrant FAM64A expression was detected in LUAD tissues by immunostaining. Taken together, our miRNA-based analysis might be effective for identifying prognostic and therapeutic molecules in LUAD.

## 1. Introduction

Lung cancer is one of the most common and lethal cancers. In 2018, approximately 2.1 million people were diagnosed with this disease, and 1.8 million patients died from it [1]. Lung cancers are divided into two pathological types: small-cell lung cancer and non-small-cell lung cancer (NSCLC). NSCLC includes squamous cell carcinoma, adenocarcinoma and large-cell carcinoma [2]. Among NSCLCs, lung adenocarcinoma (LUAD) is the most common, and it is often at an advanced stage by the time of diagnosis, and thus the prognosis of the patients is unfavorable (5-year survival rate on average below 20% on average) [3].

Recently, the survival rate of LUAD patients has improved because of the development of molecularly targeted drugs and immune checkpoint inhibitors [4,5,6]. Various molecular targeted agents have become available, based on driver gene mutations in LUAD [5,6,7]. However, there is a population of LUAD patients who harbor no driver gene mutations, indicating that several distinct molecular and genetic pathways contribute to LUAD progression.

To understand the molecular pathogenesis of LUAD, we applied a microRNA (miRNA)-based approach. miRNAs (19- to 22-nucleotide-long RNA molecules) function as fine-tuners of gene expression regulation in various cells [8,9]. A single miRNA can regulate the expression of a vast number of genes; therefore, aberrant expression of miRNAs disrupts intracellular gene expression networks. A large number of studies have shown that abnormal miRNA expression contributes to several oncogenic pathways [10,11,12,13,14].

We have previously determined miRNA expression signatures in various types of cancers using RNA sequencing [15,16,17,18,19]. Our recent studies have demonstrated that some passenger strands derived from pre-miRNAs contribute to the malignant transformation of cancer cells [15,16,17,18,19]. We have shown that both strands of miRNAs (e.g., pre-*miR-144*, pre-*miR-145* and pre-*miR-150*) were significantly downregulated in lung cancer tissues, and their ectopic expression attenuated the malignant phenotypes of lung cancer cells (e.g., cancer cell proliferation, migration and invasion) [20,21,22,23,24]. The involvement of the passenger strand of miRNAs in the pathogenesis of cancer is a new theme in cancer research.

Based on miRNA signatures by RNA sequencing, we revealed that expression levels of both strands derived from pre-*miR-99a* (*miR-99a-5p*: the guide strand; *miR-99a-3p*: the passenger strand) were suppressed in several types of cancer tissues [15,16,17,18,19]. In the current study, we investigated the tumor-suppressive functions of both strands of pre-*miR-99a* and identified their oncogenic targets in LUAD cells. Notably, a total of 19 genes (*CKS1B*, *KCMF1*, *CENPF*, *CASC5*, *MKI67*, *ESCO2*, *FANCI*, *SGOL1*, *MCM4*, *KIF11*, *NEK2*, *MTHFD2*, *NCAPG*, *RRM2*, *FAM136A*, *ZWINT*, *CDK1*, *CDKN3* and *FAM64A*) were identified as targets of pre-*miR-99a* regulation, and they significantly predicted the prognosis (5-year overall survival) of the patients with LUAD. Among the targets, we focused on *FAM64A*, as it was regulated directly by both *miR-99a-5p* and *miR-99a-3p* in LUAD cells. Our miRNA analysis strategy will accelerate the understanding of the molecular mechanism of LUAD.

## 2. Materials and Methods

### 2.1. Data Mining of miRNA Target Genes and Their Expression in LUAD Clinical Specimens

Gene expression data in LUAD obtained from The Cancer Genome Atlas (TCGA) were retrieved on 6 March 2020 from the cBioPortal database (https://www.cbioportal.org/) [25], UCSC Xena platform (https://xena.ucsc.edu/) [26] and Firebrowse (http://firebrowse.org/). The mRNA expression Z-scores and information on the clinical samples corresponding to LUAD patients were collected from cBioPortal. To categorize genes into molecular pathways based on gene set enrichment analysis (GSEA) [27], we employed the WebGestalt program (http://www.webgestalt.org/) [28].

Putative target genes possessing binding sites for *miR-99a-5p* and *miR-99a-3p* were isolated using the TargetScanHuman database ver. 7.2 (http://www.targetscan.org/vert_72/) [29]. Comprehensive correlations between mRNA and miRNA gene expression in LUAD samples from TCGA were analyzed by LinkedOmics (http://www.linkedomics.org/) [30].

### 2.2. Transfection of miRNAs, siRNAs and Plasmid Vectors into LUAD Cells and Functional Assays

The procedures for transfecting miRNAs, siRNAs and plasmid vectors were described in our previous studies [20,21,22,23,24]. Functional assays (cell proliferation and cell cycle) were performed in LUAD cells, as described in our previous studies [20,21,22,23,24]. The reagents used are listed in Supplementary Table S1.

### 2.3. Plasmid Construction and Dual-Luciferase Reporter Assays

The vectors used for this analysis were constructed as described in our previous studies [20,21,22,23,24]. Supplementary Figure S1 shows the sequences incorporated into the vectors. The analysis was performed according to our previous studies [20,21,22,23,24]. The reagents used are listed in Supplementary Table S1.

### 2.4. Immunohistochemistry

The immunohistochemistry procedure was described in our previous studies [20,21,22,23,24]. The antibodies used in this study are listed in Supplementary Table S1.

### 2.5. Stastistical Analyses

Statistical analyses were performed using GraphPad Prism 7 (GraphPad Software, La Jolla, CA, USA) and JMP Pro 14 (SAS Institute Inc., Cary, NC, USA). The Mann–Whitney U test was used to determine the significance of differences between two groups, and one-way analysis of variance and Tukey’s test for post-hoc analysis were used for multiple group comparisons. To evaluate the correlation between two variables, we applied Spearman’s rank test. Overall survival (OS) and disease-free survival (DFS) were assessed using the Kaplan–Meier method and log-rank test. To identify independent factors predicting OS and DFS, we utilized multivariate Cox proportional hazards models.

## 3. Results

### 3.1. Downregulation of miR-99a-5p and miR-99a-3p in LUAD Clinical Specimens and Their Clinical Significance

**Figure 1:**
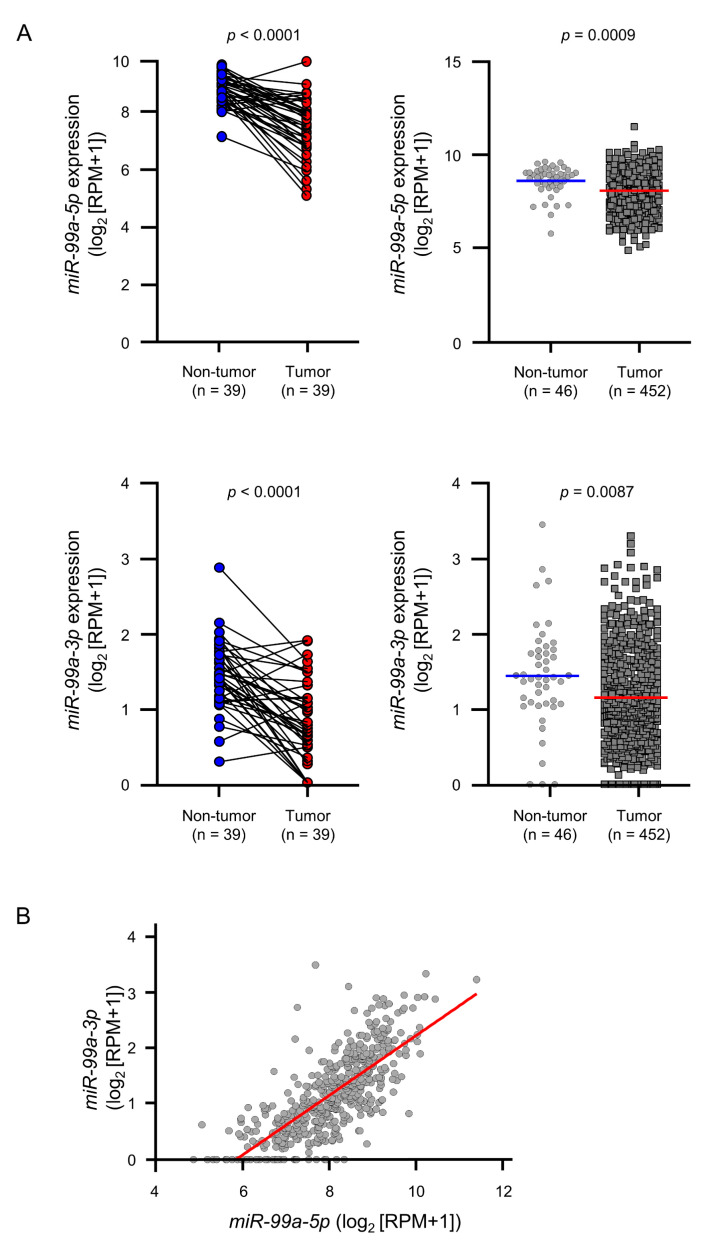
Downregulation of *miR-99a-5p* and *miR-99a-3p* in LUAD. (**A**) Comparison of the expression levels of *miR-99a-5p* and *miR-99a-3p* between tumor and non-tumor tissues in paired (left) and non-paired (right) LUAD clinical specimens from TCGA datasets. (**B**) Positive correlation between the relative expression level of *miR-99a-5p* and that of *miR-99a-3p* in clinical specimens according to Spearman’s rank tests.

The expression levels of *miR-99a-5p* and *miR-99a-3p* were evaluated using miRNA-seq data of TCGA-LUAD from Firebrowse. The miRNA-seq data showed that expression levels of *miR-99a-5p* and *miR-99a-3p* were significantly suppressed in LUAD tissues compared with normal lung tissues (Figure 1A). According to Spearman’s rank test, a positive correlation was detected between the expression levels of the two miRNA strands (*r* = 0.7716, *p* < 0.0001; Figure 1B).

**Figure 2:**
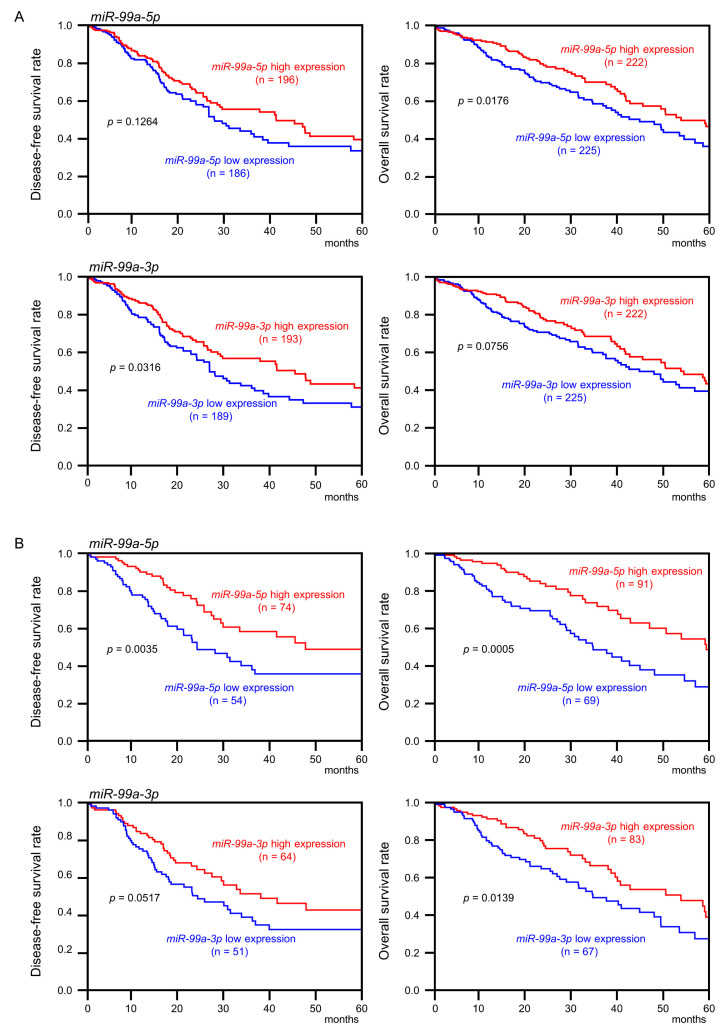
Clinical significance of *miR-99a-5p* and *miR-99a-3p* expression in LUAD. (**A**) The patients were divided into two groups according to the median expression level of *miR-99a-5p* or *miR-99a-3p*: high (red lines) and low (blue lines) expression groups. (**B**) The patients were divided into two groups, top 25% and low 25%. High expression of *miR-99a-5p* and *miR-99a-3p* is represented by red lines; low expression of these miRNAs is represented by blue lines.

Kaplan–Meier plot and log-rank test using survival data from TCGA-LUAD revealed that low expression of *miR-99a-5p* was associated with a worse prognosis compared with high expression (DFS: *p* = 0.1264; OS: *p* = 0.00176) (Figure 2A). Similarly, low expression of *miR-99a-3p* was associated with a worse prognosis compared with high expression (DFS: *p* = 0.0316; OS: *p* = 0.0756) (Figure 2A).

Similarly, the patients were divided into two groups according to the expression levels of *miR-99a-5p* and *miR-99a-3p* (top 25%: red lines and low 25%: blue lines) and analyzed. Kaplan–Meier plot and log-rank test showed that low expression of *miR-99a-5p* was associated with a worse prognosis compared with high expression (DFS: *p* = 0.0035; OS: *p* = 0.0005) (Figure 2B). Low expression of *miR-99a-3p* was associated with a worse prognosis compared with high expression (DFS: *p* = 0.0517; OS: *p* = 0.0139) (Figure 2B).

### 3.2. Tumor-Suppressive Functions of miR-99a-5p and miR-99a-3p Assessed by Ectopic Expression Assays

**Figure 3:**
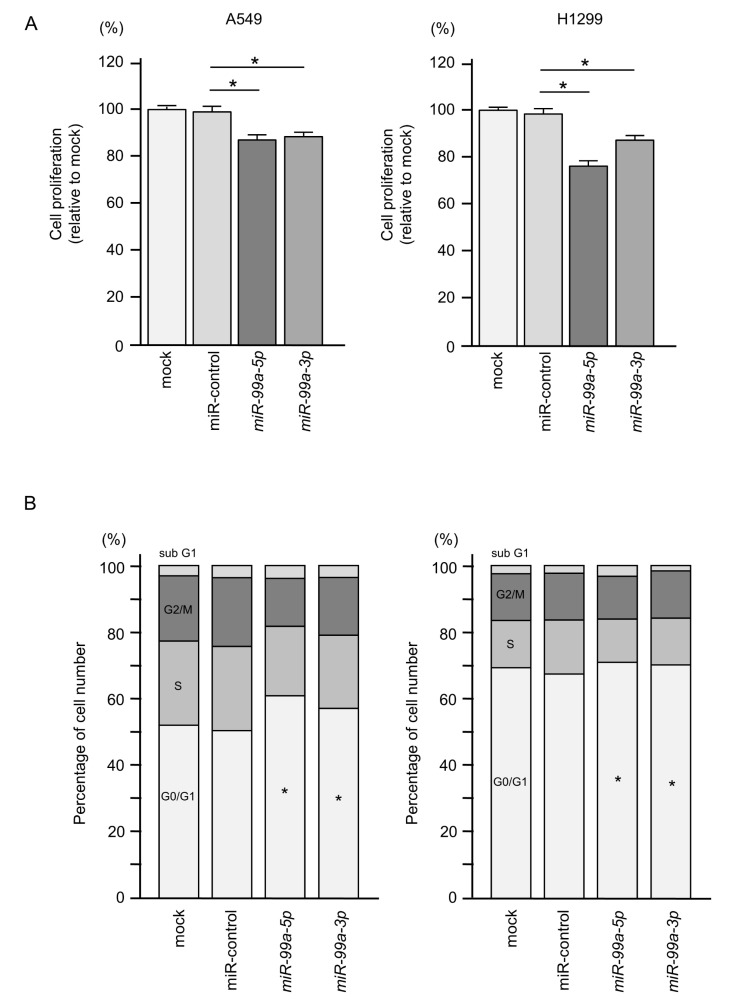
Functional assays of cell proliferation and cell cycle arrest following ectopic expression of *miR-99a-5p* or *miR-99a-3p* in LUAD cell lines (A549 and H1299 cells). (**A**) Cell proliferation assessed using XTT assays at 72 h after miRNA transfection (* *p* < 0.0001). (**B**) Flow cytometric analysis of the cell cycle phase distribution of control cells and cells transfected with *miR-99a-5p* or *miR-99a-3p*. Cells were evaluated at 72 h after miRNA transfection (* *p* < 0.0001).

We assessed changes in cell proliferation and cell cycle after ectopic expression of these miRNAs into A549 and H1299 cells. Cell proliferation (XTT assay) was significantly inhibited by *miR-99a-5p* or *miR-99a-3p* expression in A549 and H1299 LUAD cell lines (Figure 3A). To investigate the synergistic effects of *miR-99a-5p* and *miR-99a-3p*, we performed proliferation assays with co-transfection of *miR-99a-5p* and *miR-99a-3p* in LUAD cells (A549 and H1299), but they did not show synergistic effects of these miRNAs transfection (Supplementary Figure S2). In the cell cycle analysis by flow cytometry, the number of LUAD cells in the G0/G1 phase was increased after ectopic expression of these miRNAs compared with control miRNA (Figure 3B). Our data suggest that ectopic expression of *miR-99a-5p* and *miR-99a-3p* induces G1 arrest in LUAD cells.

### 3.3. Identification of miR-99a-5p and miR-99a-3p Target Genes in LUAD

**Figure 4:**
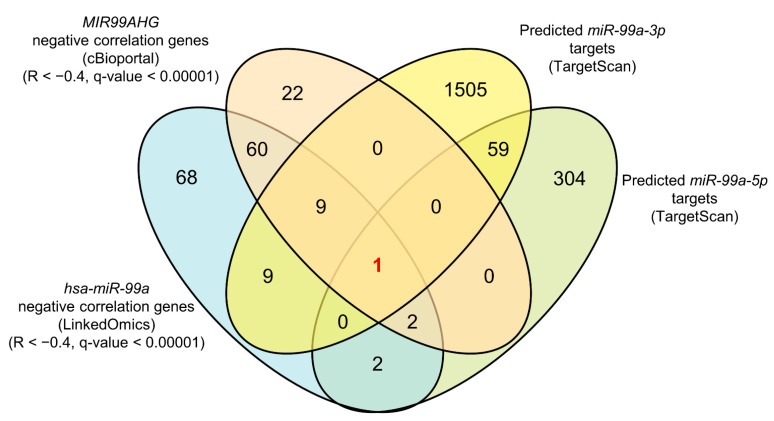
Identification of putative targets of *miR-99a-5p* or *miR-99a-3p* regulation in LUAD cells. Prediction of *miR-99a-5p* and *miR-99a-3p* target genes using the TargetScanHuman, cBioportal and LinkedOmics databases. Venn diagrams represent the number of putative target genes regulated by *miR-99a-5p* or *miR-99a-3p*.

To identify genes regulated by pre-*miR-99a* in LUAD cells, we applied in silico analyses using the TargetScanHuman (release 7.2), LinkedOmics and cBioportal databases (Figure 4). A total of 23 genes were identified as pre-*miR-99a* targets in LUAD cells (five *miR-99a-5p* targets and 19 *miR-99a-3p* targets; Table 1). Notably, *FAM64A* was identified as a target of both *miR-99a-5p* and *miR-99a-3p*.

### 3.4. Clinical Significance of miR-99a-5p and miR-99a-3p Target Genes in LUAD Pathogenesis

**Figure 5:**
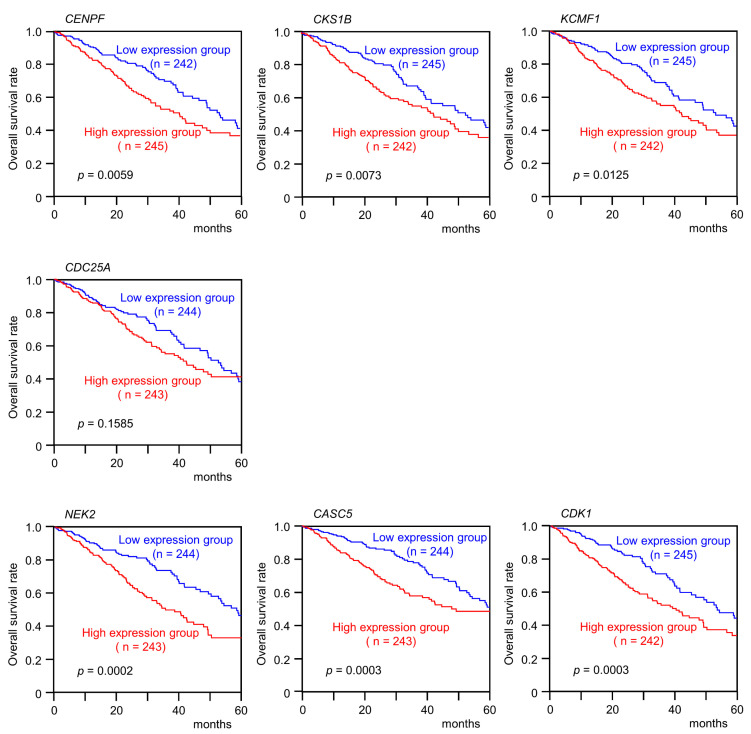

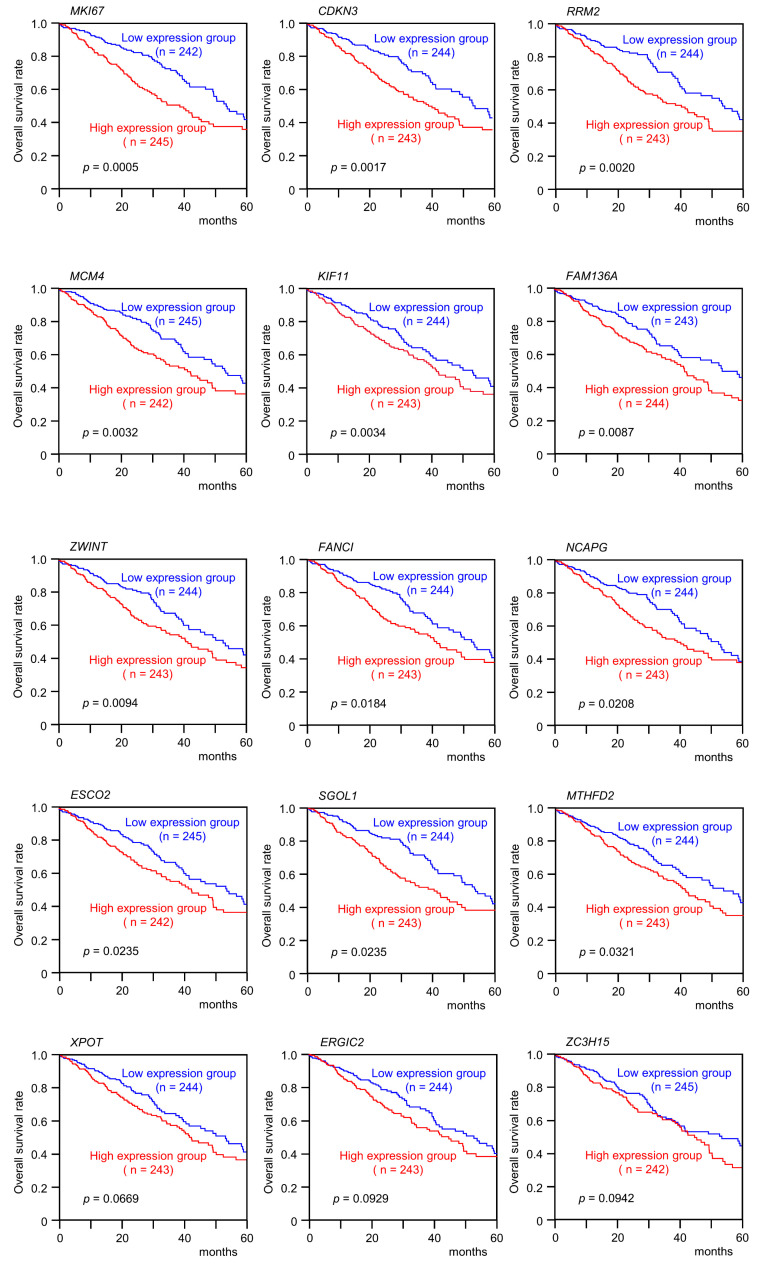
Clinical significance of pre-*miR-99a* target genes in TCGA database. Kaplan–Meier survival curves and log-rank comparisons of patients with LUAD using TCGA datasets. Patients were divided into two groups according to the median expression of each pre-*miR-99a* target gene evaluated: high and low expression groups. The red and blue lines represent the high and low expression groups, respectively. High expression mRNA of 18 genes (*CKS1B*, *KCMF1*, *CENPF*, *CASC5*, *MKI67*, *ESCO2*, *FANCI*, *SGOL1*, *MCM4*, *KIF11*, *NEK2*, *MTHFD2*, *NCAPG*, *RRM2*, *FAM136A*, *ZWINT*, *CDK1* and *CDKN3*) significantly predicted worse survival (5-year overall survival rate) in patients with LUAD. The expression data were downloaded from http://www.oncolnc.org.

**Figure 6:**
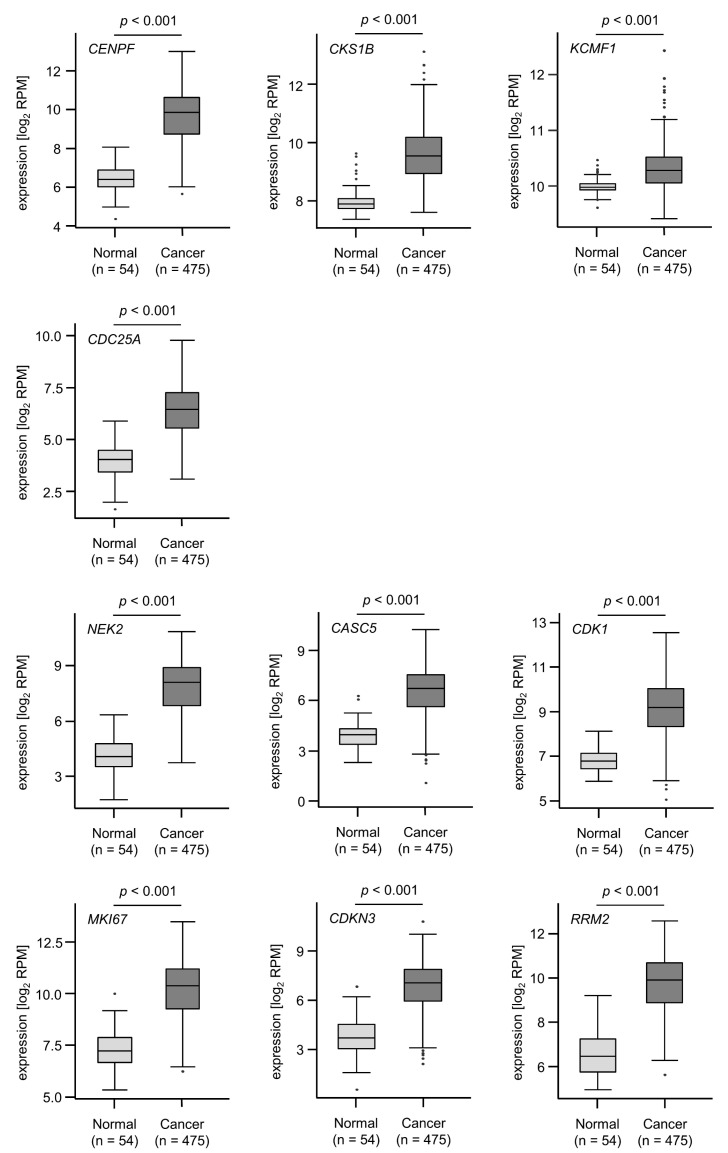

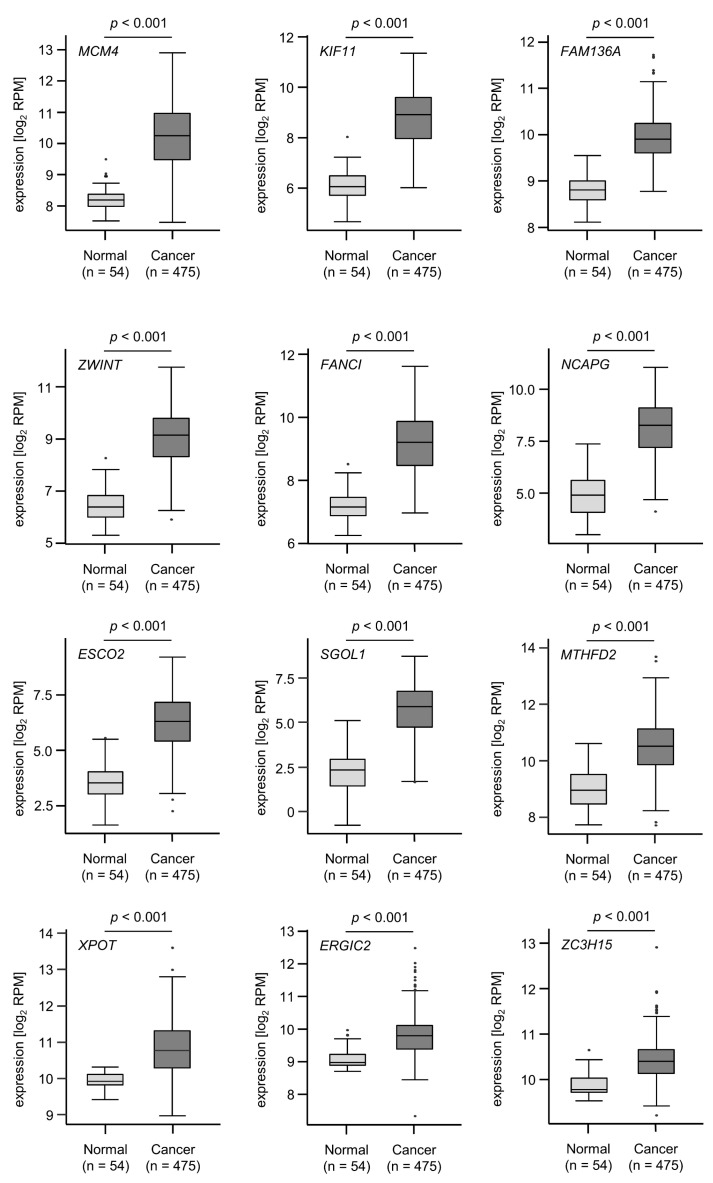
Expression levels of pre-*miR-99a* target genes in LUAD clinical specimens. Using TCGA datasets, the expression levels of all 22 pre-*miR-99a* target genes evaluated were upregulated in LUAD clinical specimens (*n* = 475) compared with normal lung tissues (*n* = 54). The expression data were downloaded from http://firebrowse.org/.

We evaluated the associations between expression levels of the genes and survival using TCGA and GEO datasets. Among 22 of the 23 target genes (excluding *FAM64A*), high expression of 18 genes (*CKS1B*, *KCMF1*, *CENPF*, *CASC5*, *MKI67*, *ESCO2*, *FANCI*, *SGOL1*, *MCM4*, *KIF11*, *NEK2*, *MTHFD2*, *NCAPG*, *RRM2*, *FAM136A*, *ZWINT*, *CDK1* and *CDKN3*) significantly predicted worse survival (OS: 5-year survival rate) in patients with LUAD (Figure 5). We also adjusted the multiplicity by using Benjamini–Hochberg analysis and confirmed that 19 out of these 23 target genes were significant (Supplementary Table S3). All genes were upregulated in cancer tissues compared with normal tissues (Figure 6). We also classified these target genes according to Gene Ontology (GO: Biological Process) criteria by using the GeneCodis database. The GO classification of the genes controlled by each miRNA was shown in Supplementary Table S4. Each miRNA has regulated genes associated with “cell cycle (GO: 0007049)” and “cell division (GO: 0051301)”.

### 3.5. Clinical Significance of FAM64A in LUAD Pathogenesis

**Figure 7:**
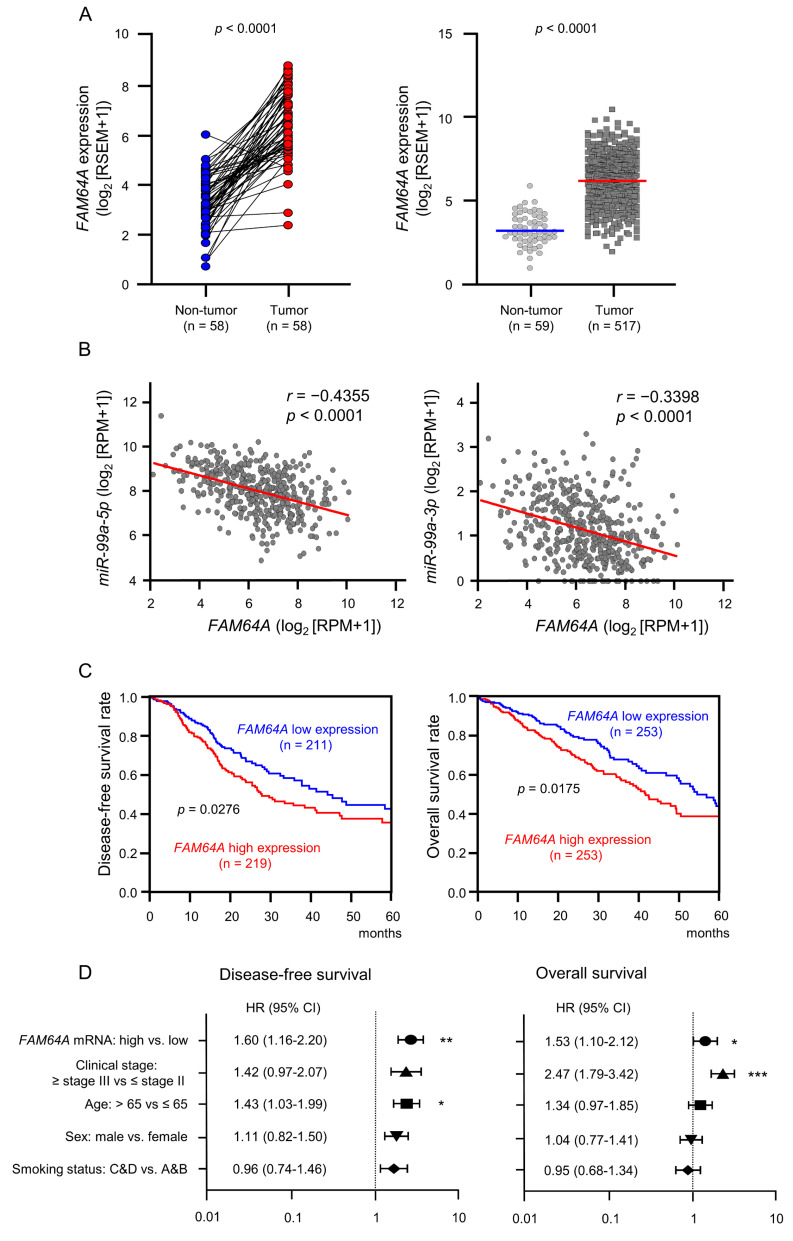
Clinical significance of *FAM64A* expression in LUAD. (**A**) Comparison of *FAM64A* expression levels between tumor and non-tumor tissues in paired (left) and non-paired (right) LUAD clinical specimens from TCGA datasets. Upregulation of *FAM64A* was detected in LUAD tissues. (**B**) Correlations between the relative expression level of *FAM64A* and that of *miR-99a-5p* or *miR-99a-3p*. Spearman’s rank test showed a negative correlation between *FAM64A* and *miR-99a-5p* or *miR-99a-3p* expression levels in clinical specimens. (**C**) Kaplan–Meier survival curves and log-rank comparisons of patients with LUAD using TCGA database. Patients were divided into two groups according to the median *FAM64A* expression level: high and low expression groups. The red and blue lines represent the high and low expression groups, respectively. (**D**) Forest plot of the multivariate analysis results assessing independent prognostic factors for disease-free and overall survival, including *FAM64A* expression (high vs. low) (* *p* < 0.05, ** *p* < 0.01, *** *p* < 0.001).

**Figure 8:**
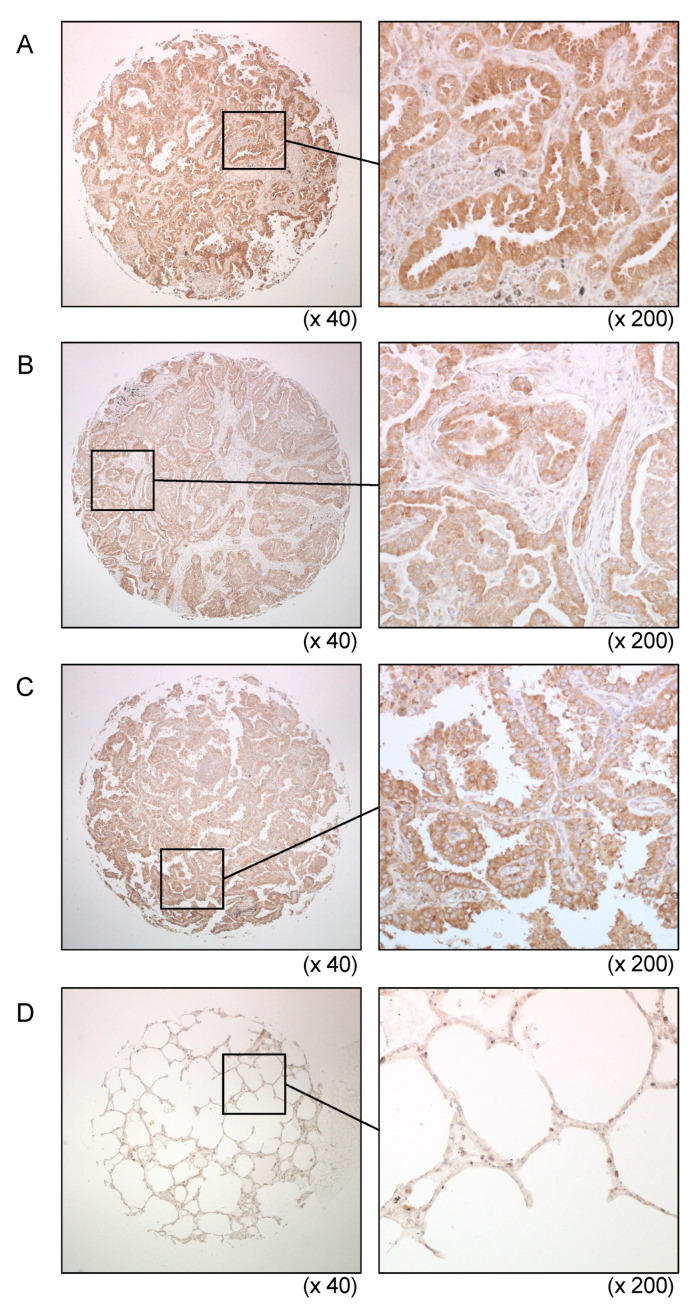
Overexpression of FAM64A in LUAD clinical specimens. (**A**–**C**) Immunohistochemical staining of FAM64A in LUAD tissues. Overexpression of FAM64A was detected in the cytoplasm and/or nuclei of cancer cells. On the other hand, expression of FAM64A was low in normal lung cells (**D**).

Overexpression of *FAM64A* in LUAD tissues was confirmed by RNA-seq data from TCGA-LUAD (Figure 7A). Spearman’s rank test indicated negative correlations of *FAM64A* expression with both *miR-99a-5p* and *miR-99a-3p* expression (Figure 7B). We investigated the clinical significance of *FAM64A* expression in LUAD patients using the TCGA database. High expression of *FAM64A* was associated with a significantly poor prognosis compared with low expression (DFS: *p* = 0.0276; OS: *p* = 0.0175; Figure 7C) and was identified as an independent prognostic factor of survival in the multivariate analysis (*p* < 0.01; Figure 7D). FAM64A protein expression was also evaluated in LUAD clinical specimens using immunohistochemistry. Overexpression of FAM64A protein was detected in cancer lesions in LUAD clinical specimens (Figure 8).

### 3.6. Direct Regulation of FAM64A by miR-99a-5p and miR-99a-3p in LUAD Cells

**Figure 9:**
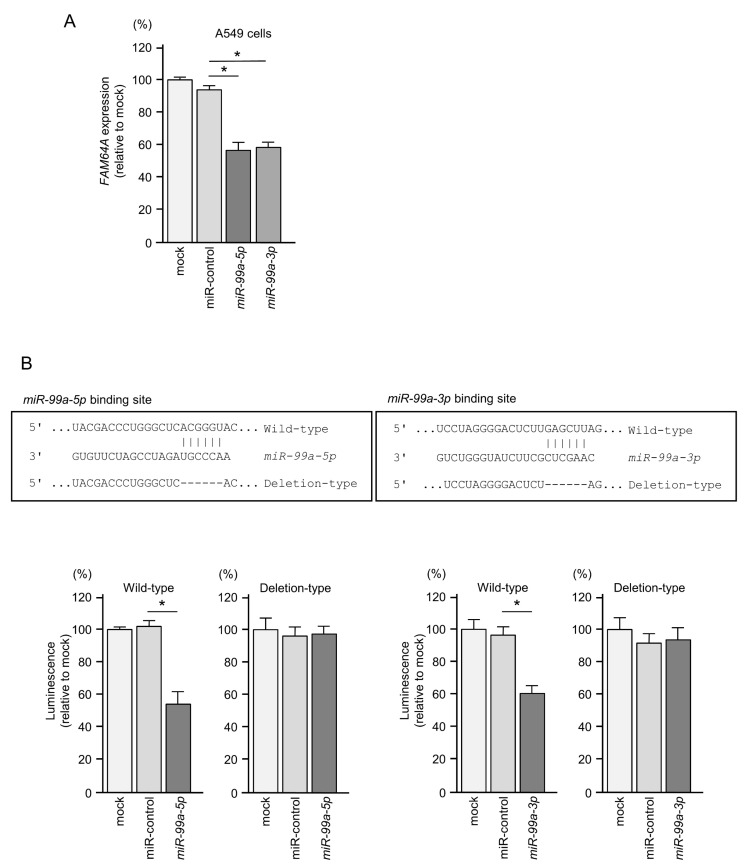
Direct regulation of *FAM64A* by *miR-99a-5p* and *miR-99a-3p* in LUAD cells. (**A**) Significantly reduced expression of *FAM64A* mRNA by *miR-99s-5p* or *miR-99a-3p* transfection in A549 cells (at 72 h after transfection; * *p* < 0.0001). (**B**) Predictions of *miR-99a*-binding sites using TargetScanHuman database analyses. Each miRNA strand (*miR-99a-5p* and *miR-99a-3p*) had one binding site in the 3′UTR of *FAM64A.* Dual-luciferase reporter assays showed that luminescence activity was reduced by co-transfection of the *FAM64A* wild-type vector (containing the *miR-99a-5*p-binding site) with *miR-99a-5p* or of the *FAM64A* wild-type vector (containing the *miR-99a-3*p-binding site) with *miR-99a-3p* in A549 cells. Normalized data were calculated as *Renilla*/firefly luciferase activity ratios (* *p* < 0.0001).

We focused on *FAM64A* because its expression was found to be controlled by both strands of pre-*miR-99a* (*miR-99a-5p* and *miR-99a-3p*). The expression level of *FAM64A* was significantly reduced after transfection of *miR-99a-5p* and *miR-99a-3p* in LUAD cells (Figure 9A).

There is one miRNA-binding site for each miRNA strand (*miR-99a-5p* and *miR-99a-3p*) in the 3′UTR region of *FAM64A* (Figure 9B). In dual-luciferase reporter assays, luciferase activity was significantly decreased by co-transfection of *miR-99a-5p* or *miR-99a-3p* with the vector containing the wild-type 3′UTR of *FAM64A* in A549 cells. On the other hand, the transfection of the deletion vector (containing the deletion-type 3′UTR of *FAM64A*) prevented this decrease in luminescence (Figure 9B), suggesting that *miR-99a-5p* and *miR-99a-3p* bind directly to the 3′UTR of *FAM64A* in LUAD cells.

### 3.7. Effects of FAM64A Knockdown on Cell Proliferation and Cell Cycle in LUAD Cells

**Figure 10:**
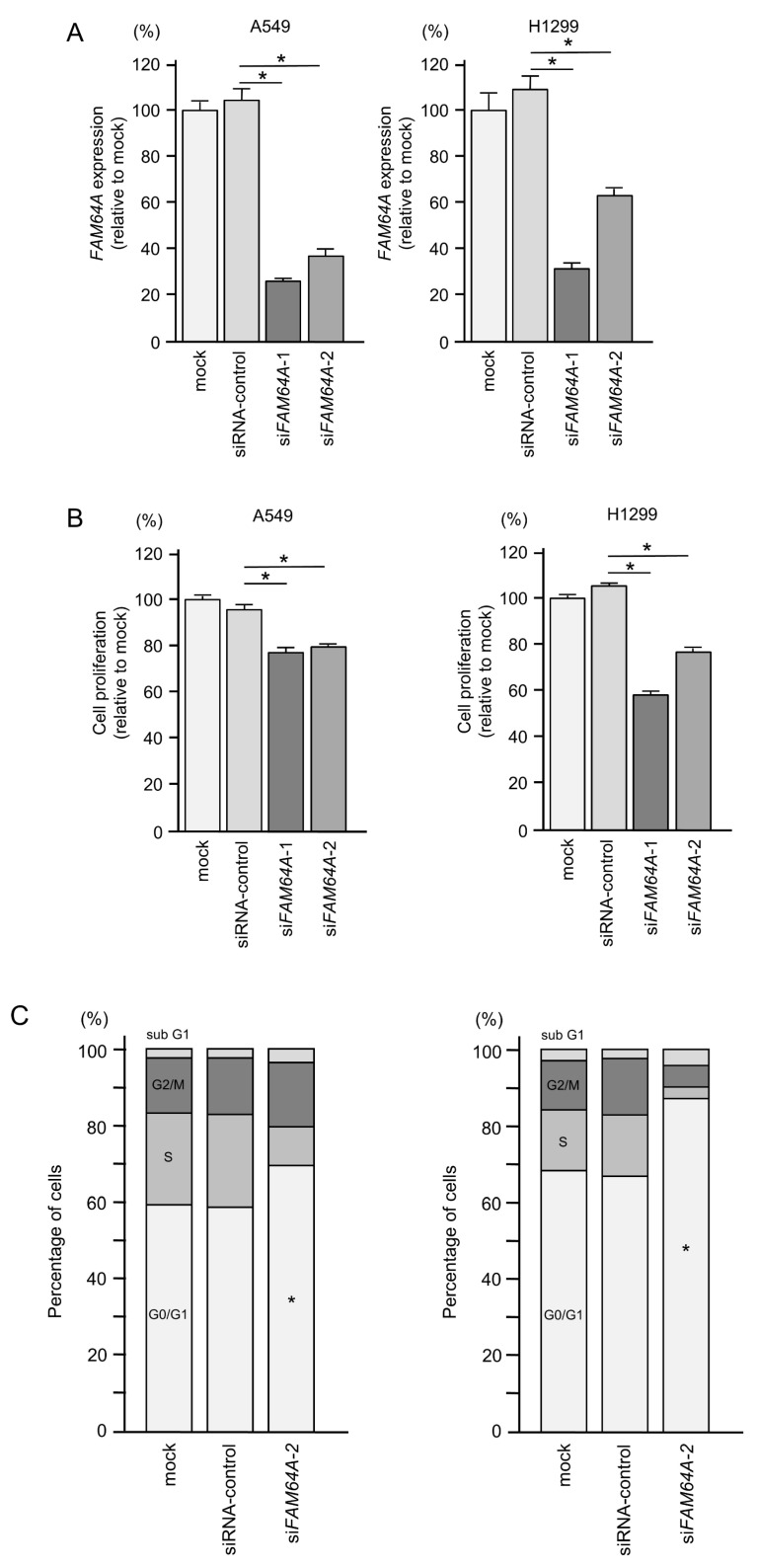
Effect of *FAM64A* knockdown on cell proliferation and cell cycle arrest in LUAD cells. (**A**) Successful suppression of *FAM64A* expression by si*FAM64A*-1 or si*FAM64A*-2 transfection in A549 and H1299 cells. (**B**) Cell proliferation assessed by XTT assay at 72 h after miRNA transfection (* *p* < 0.0001). (**C**) Flow cytometric analyses of cell cycle phase distributions in control cells and cells transfected with si*FAM64A*. The cells were assessed at 72 h after miRNA transfection (* *p* < 0.0001).

To investigate the oncogenic function of *FAM64A* in LUAD cells, we performed knockdown assays using siRNAs. The expression level of *FAM64A* was successfully reduced by two different siRNAs (si*FAM64A*-1 and si*FAM64A*-2; Figure 10A).

The proliferation of LUAD cells was attenuated by the transfection of each si*FAM64A* (Figure 10B).

Cell cycle assays demonstrated that the number of LUAD cells in the G0/G1 phase was increased after knockdown of *FAM64A* (Figure 10C). These data indicate that the expression of *FAM64A* enhances cell cycle progression.

### 3.8. FAM64A Effects on Molecular Pathways in LUAD

We identified differentially expressed genes from TCGA-LUAD RNA-seq between *FAM64A* high expression group and low expression group.

**Figure 11:**
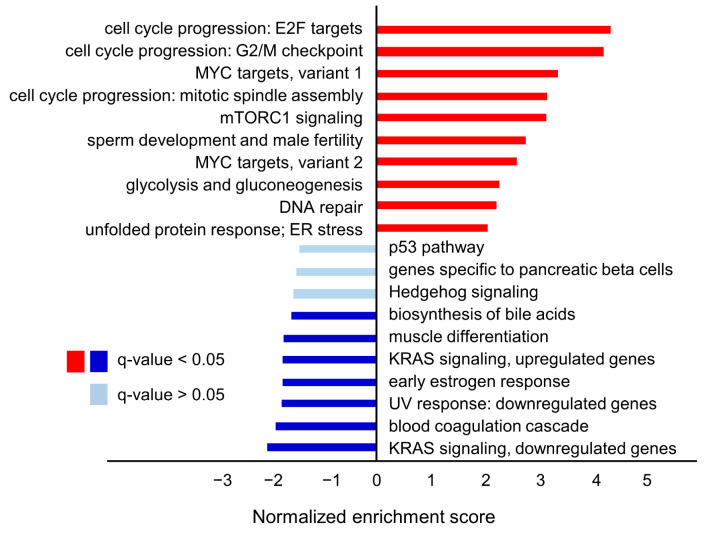

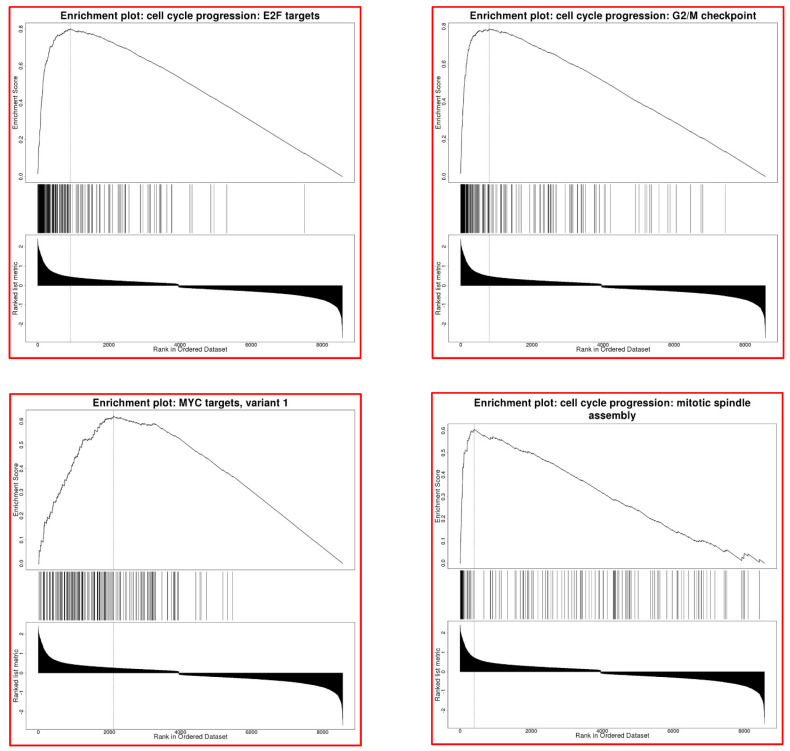
TCGA database analysis of the clinical significance and function of *FAM64A* in LUAD clinical specimens. The bar graph shows the results of gene set enrichment analysis (GSEA) of the genes differentially expressed between high and low *FAM64A* expression groups in LUAD patients. Four representative GSEA plots are shown below for E2F targets, G2/M checkpoint, MYC target 1 variant 1 and mitotic spindle assembly with q-values < 0.05. These pathway terms were significantly enriched in the high *FAM64A* expression group.

GSEA showed that the top signaling pathways enriched in the high *FAM64A* expression group were cell cycle-associated terms, such as E2F targets, G2M checkpoints, MYC targets and mitotic spindle assembly (Figure 11).

**Figure 12:**
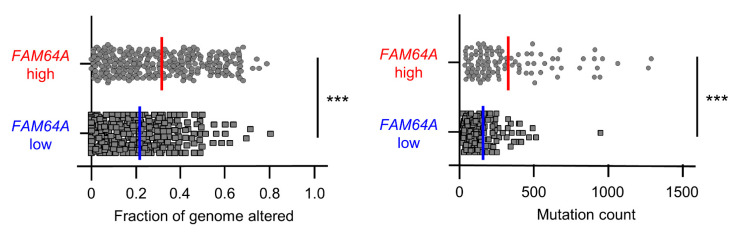
Associations of genome alterations and mutation counts with *FAM64A* expression in LUAD clinical specimens. Proportion of genome alterations (percentage of chromosome regions with copy number alterations relative to all regions evaluated; (**left**)) and the mutation count (number of mutational events per case; (**right**)) were significantly increased in the high compared with the low *FAM64A* expression group (*** *p* < 0.001).

Finally, we found that the proportion of genome alterations (percentage of chromosome regions with copy number alterations relative to all regions evaluated) and the mutation count (the number of mutational events per case) were significantly increased in the high *FAM64A* expression group (Figure 12), suggesting that *FAM64A* expression may be associated with genetic mutations and genomic instability in LUAD cells.

## 4. Discussion

Active genomic research has led to the discovery of driver genes/mutations critical to lung cancer [31]. Molecularly targeted drugs were developed based on these driver genes, and the prognosis of advanced LUAD has greatly improved due to the emergence of molecularly targeted therapeutic agents [32]. However, even with these therapeutic agents, it is difficult to eliminate cancer cells from patients. Continued exploration of molecular networks in LUAD cells provides useful information for developing novel therapeutics.

To identify novel therapeutic targets and pathways, we have previously identified tumor-suppressive miRNAs and their oncogenic targets in LUAD [22,23,24]. A feature of our study is that we analyzed both strands of pre-miRNAs: the guide and passenger strands. The general theory regarding miRNA biogenesis so far is that the passenger strand of a miRNA derived from a pre-miRNA is decomposed in the cytoplasm and has no function [9,10]. Contrary to this belief, recent reports have shown that some passenger strands of miRNAs regulate oncogenes in cancer cells and exert tumor-suppressive functions [33]. Our recent studies demonstrated that some passenger strands of miRNAs, e.g., *miR-143-5p*, *miR-145-3p* and *miR-150-3p*, behave as tumor-suppressive miRNAs in LUAD cells by targeting oncogenes, e.g., *LMNB2*, *MCM4* and *TNS4*, respectively [22,23,24].

In lung cancer, several studies have shown that *miR-99a-5p* acts as a tumor-suppressive miRNA by targeting critical oncogenic pathways, including AKT1 and mTOR signaling [34,35]. In contrast, there are few reports on *miR-99a-3p* function in lung cancer cells. Based on our miRNA signatures, we showed that *miR-99a-3p* also acts as a tumor-suppressive miRNA in prostate cancer and head and neck squamous cell carcinoma [36,37]. Of particular interest in those papers is that many of the genes identified as targets of *miR-99a-3p* contribute to malignant phenotypes of cancer cells and significantly predict the worse prognosis of the patients [37]. The search for genes regulated by the passenger strands of miRNAs will provide new information for exploring the molecular mechanisms of LUAD.

In this study, a total of 19 genes (*CKS1B*, *KCMF1*, *CENPF*, *CASC5*, *MKI67*, *ESCO2*, *FANCI*, *SGOL1*, *MCM4*, *KIF11*, *NEK2*, *MTHFD2*, *NCAPG*, *RRM2*, *FAM136A*, *ZWINT*, *CDK1*, *CDKN3* and *FAM64A*) identified as pre-*miR-99a* targets appear to be intimately involved in LUAD pathogenesis. Interestingly, many of these genes are involved in the cell cycle, cell division and chromosome segregation. These molecules are essential for cell division and may be potential targets for cancer drug development. For example, KIF11 is a kinesin, a microtubule-based motor protein that mediates diverse intracellular functions, such as its critical roles in cell division and intercellular vesicle and organelle transport [38,39]. Several inhibitors of KIF11 have entered phase I and II clinical trials [39]. Functional analyses of the genes regulated by pre-*miR-99a* are useful for exploring molecular networks in LUAD.

In this study, we focused on *FAM64A* because its expression is regulated by both strands of pre-*miR-99a* (*miR-99a-5p* and *miR-99a-3p*) in LUAD cells. FAM64A (also known as *PIMREG*, *CAKM*, *CATS* and *RCS1*) was initially identified as a CALM/PICALM-interacting protein using a yeast two-hybrid system [40]. The fusion protein CALM/AF10, t(10;11)(p13;q14), plays a crucial role in acute myeloid leukemia, acute lymphoblastic leukemia and malignant lymphoma [41,42]. Previous studies demonstrated that *FAM64A* contributes to cell cycle progression [43,44,45]. Overexpression of *FAM64A* was reported in leukemia, lymphoma and several types of solid cancer [46]. In breast cancer, overexpression of FAM64A enhanced the transactivation of NF-κB by disrupting the NF-κB/IκBα negative feedback loop [47]. Another study demonstrated that FAM64A regulates STAT3 activation and is involved in Th17 differentiation, colitis and colorectal cancer development [48]. These findings indicate that *FAM64A* behaves as a transcriptional regulator contributing to cell cycle progression. *FAM64A* might be a potential prognostic factor and therapeutic target in LUAD.

## 5. Conclusions

Both the guide (*miR-99a-5p*) and passenger (*miR-99a-3p*) strands of pre-*miR-99a* showed antitumor functions in LUAD cells. A total of 23 genes were identified as putative pre-*miR-99a* targets in LUAD cells. Among these targets, 19 genes (*CKS1B*, *KCMF1*, *CENPF*, *CASC5*, *MKI67*, *ESCO2*, *FANCI*, *SGOL1*, *MCM4*, *KIF11*, *NEK2*, *MTHFD2*, *NCAPG*, *RRM2*, *FAM136A*, *ZWINT*, *CDK1*, *CDKN3* and *FAM64A*) were closely associated with the molecular pathogenesis of LUAD. *FAM64A* was directly regulated by both strands of pre-*miR-99a*, and its aberrant expression enhanced cancer cell proliferation.

## Figures and Tables

**Table 1:** Putative targets by *miR-99a-5p* and *miR-99a-3p* regulation in LUAD cells.

Entrez Gene ID	Gene Symbol	Gene Name	Location	Total Sites	5-Year OS *p*-Value
Putative targets by *miR-99a-5p* regulation in LUAD cells					
1063	*CENPF*	Centromere protein F	1q41	1	0.0059
1163	*CKS1B*	CDC28 protein kinase regulatory subunit 1B	1q21.3	1	0.0073
56888	*KCMF1*	Potassium channel modulatory factor 1	2p11.2	1	0.0125
417685	*FAM64A*	Family with sequence similarity 64 member A	17p13.2	1	0.0176
993	*CDC25A*	Cell division cycle 25A	3p21.31	1	0.1585
Putative targets by *miR-99a-3p* regulation in LUAD cells					
4751	*NEK2*	NIMA related kinase 2	1q32.3	1	0.0002
57082	*CASC5*	Cancer susceptibility candidate 5	15q15.1	1	0.0003
983	*CDK1*	Cyclin dependent kinase 1	10q21.2	1	0.0003
4288	*MKI67*	Marker of proliferation Ki-67	10q26.2	1	0.0005
1033	*CDKN3*	Cyclin dependent kinase inhibitor 3	14q22.2	1	0.0017
6241	*RRM2*	Ribonucleotide reductase regulatory subunit M2	2p25.1	1	0.002
4173	*MCM4*	Minichromosome maintenance complex component 4	8q11.21	1	0.0032
3832	*KIF11*	Kinesin family member 11	10q23.33	1	0.0034
84908	*FAM136A*	Family with sequence similarity 136 member A	2p13.3	1	0.0087
11130	*ZWINT*	ZW10 interacting kinetochore protein	10q21.1	1	0.0094
55215	*FANCI*	FA complementation group I	15q26.1	1	0.0108
64151	*NCAPG*	Non-SMC condensin I complex subunit G	4p15.31	1	0.0208
157570	*ESCO2*	Establishment of sister chromatid cohesion N-acetyltransferase 2	8p21.1	1	0.0235
151648	*SGOL1*	Shugoshin 1	3p24.3	1	0.0235
10797	*MTHFD2*	Methylenetetrahydrofolate dehydrogenase (NADP+ dependent) 2, methenyltetrahydrofolate cyclohydrolase	2p13.1	1	0.0321
11260	*XPOT*	Exportin for tRNA	12q14.2	1	0.0669
417685	*FAM64A*	Family with sequence similarity 64 member A	17p13.2	1	0.0756
51290	*ERGIC2*	ERGIC and golgi 2	12p11.22	1	0.0929
55854	*ZC3H15*	Zinc finger CCCH-type containing 15	2q32.1	1	0.0942

## Supplementary Materials

The following are available online at https://www.mdpi.com/2073-4409/9/9/2083/s1, Figure S1: Vector inserted sequences; Figure S2: Effects of ectopic expression of *miR-99a-5p* and *miR-99a-3p* on LUAD cells; Table S1: Reagent used in this study; Table S2: Characteristics of the patients used in immunostaining; Table S3: 23 target genes analyzed by Benjamini-Hochberg method; Table S4: Significantly enriched annotations regulated by *miR-99a-5p*/*miR-99a-3p* in LUAD cells and pathways regulated by *miR-99a-5p*/*miR-99a-3p* in LUAD cells.
