# Supplementary material for: FAM64A: A Novel Oncogenic Target of Lung Adenocarcinoma Regulated by Both Strands of miR-99a (miR-99a-5p and miR-99a-3p)
Source: Cells. 2020 Sep 11;9(9):2083. doi: 10.3390/cells9092083 (PMC7564711; doi:10.3390/cells9092083)
Supplement: Supplementary file 1 [file cells-09-02083-s001.pdf]

cells

*miR-99a-5p* wild-type vector
gatatttccaggcttacgaccctgggctcacgggtacctatttatatg

*miR-99a-5p* deletion-type vector
gatatttccaggcttacgaccctgggctc------acctatttatatgctcagtg

*miR-99a-3p* wild-type vector
ttctgctttcctaggggactcttgagcttagaaactcatcgtacacttg

*miR-99a-3p* deletion-type vector
ttctgctttcctaggggactctt------agaaactcatcgtacacttgacct

**Figure S1.** Vector inserted sequences.

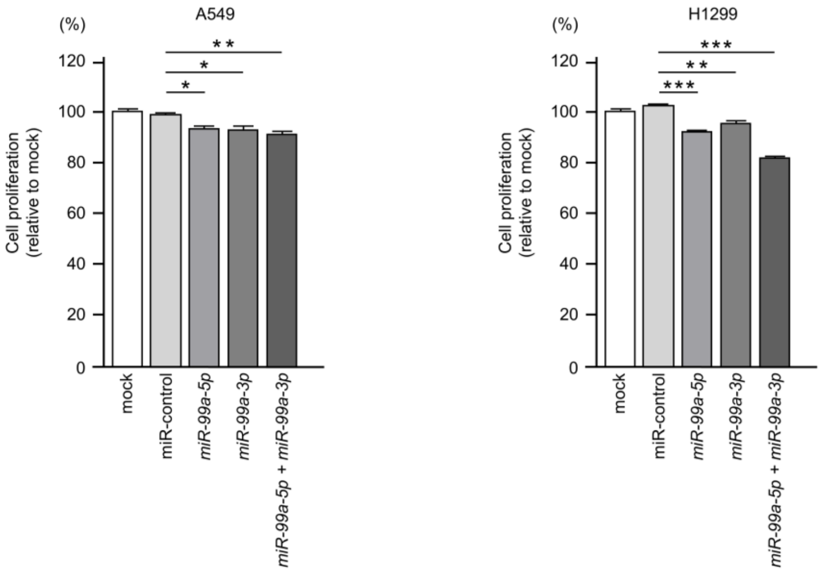


**Figure 2.** Effects of ectopic expression of *miR-99a-5p* and *miR-99a-3p* on LUAD cells. Cell proliferation was determined by XTT assays 72 h following transfection with *miR-99a-5p* (5nM), *miR-99a-3p* (5nM) or *miR-99a-5p* (5nM) + *miR-99a-3p* (5nM) (**p* < 0.05, ***p* < 0.01, ****p* < 0.0001).

**Table S1.** Reagent used in this study.

| Antibody | Dilution | Catalog number | Company |
|---|---|---|---|
| FAM64A | IHC 1:100 | ab251896 | Abcam, Cambridge, UK |

| miRNA species | Concentration | Assay ID | Company |
|---|---|---|---|
| *miR-99a-5p* | 10 nM | PM 10719 | Applied Biosystems, Foster City, CA, USA |
| *miR-99a-3p* | 10 nM | PM 12983 | Applied Biosystems, Foster City, CA, USA |
| anti-miR Negative Control #1 | 10 nM | AM 17010 | Applied Biosystems, Foster City, CA, USA |

| siRNA | Concentration | Catalog number | Company |
|---|---|---|---|
| si*FAM64A* | 10 nM | HSS147670 and HSS147671 | Invitrogen, Carlsbad, CA, USA |

| primer and probe | | Assay ID | Company |
|---|---|---|---|
| *FAM64A* | | Hs00760463_m1 | Applied Biosystems, Foster City, CA, USA |
| *GUSB* | | Hs00939627_m1 | Applied Biosystems, Foster City, CA, USA |

| Plasmid vector | | Catalog number | Company |
|---|---|---|---|
| psiCHECK-2 vector | | C8021 | Promega, Madison, WI, USA |

**Table S2.** Characteristics of the patients used in immunostaining.

| Patients | Age | Sex | T | N | M | Pathological stage | Type |
|---|---|---|---|---|---|---|---|
| A | 47 | M | 2 | 0 | 0 | IB | LUAD |
| B | 56 | F | 2 | 1 | 0 | IIA | LUAD |
| C | 67 | M | 2 | 0 | 0 | IB | LUAD |
| D | 16 | F | - | - | - | - | Normal lung tissue |

LUAD: lung adenocarcinoma

**Table S3.** 23 target genes analyzed by Benjamini-Hochberg method.

| Entrez GeneID | Gene symbol | *p*-value | i | qi=pi*N/i | FDR threshold | Judgement |
|---|---|---|---|---|---|---|
| 983 | *CDK1* | 0.0003 | 3 | 0.0023 | 0.05 | Significant |
| 4288 | *MKI67* | 0.0005 | 4 | 0.0029 | 0.05 | Significant |
| 57082 | *CASC5* | 0.0003 | 2 | 0.0035 | 0.05 | Significant |
| 4751 | *NEK2* | 0.0002 | 1 | 0.0046 | 0.05 | Significant |
| 6241 | *RRM2* | 0.002 | 6 | 0.0077 | 0.05 | Significant |
| 1033 | *CDKN3* | 0.0017 | 5 | 0.0078 | 0.05 | Significant |
| 3832 | *KIF11* | 0.0034 | 8 | 0.0098 | 0.05 | Significant |
| 4173 | *MCM4* | 0.0032 | 7 | 0.0105 | 0.05 | Significant |
| 1063 | *CENPF* | 0.0059 | 9 | 0.0151 | 0.05 | Significant |
| 1163 | *CKS1B* | 0.0073 | 10 | 0.0168 | 0.05 | Significant |
| 11130 | *ZWINT* | 0.0094 | 12 | 0.0180 | 0.05 | Significant |
| 84908 | *FAM136A* | 0.0087 | 11 | 0.0182 | 0.05 | Significant |
| 55215 | *FANCI* | 0.0108 | 13 | 0.0191 | 0.05 | Significant |
| 56888 | *KCMF1* | 0.0125 | 14 | 0.0205 | 0.05 | Significant |
| 417685 | *FAM64A* | 0.0175 | 15 | 0.0268 | 0.05 | Significant |
| 64151 | *NCAPG* | 0.0208 | 16 | 0.0299 | 0.05 | Significant |
| 151648 | *SGOL1* | 0.0235 | 18 | 0.0300 | 0.05 | Significant |
| 157570 | *ESCO2* | 0.0235 | 17 | 0.0318 | 0.05 | Significant |
| 10797 | *MTHFD2* | 0.0321 | 19 | 0.0389 | 0.05 | Significant |
| 11260 | *XPOT* | 0.0669 | 20 | 0.0769 | 0.05 | NS |
| 55854 | *ZC3H15* | 0.0942 | 22 | 0.0985 | 0.05 | NS |
| 51290 | *ERGIC2* | 0.0929 | 21 | 0.1017 | 0.05 | NS |
| 993 | *CDC25A* | 0.1585 | 23 | 0.1585 | 0.05 | NS |

NS: not significant

**Table S4A.** Significantly enriched annotations regulated by *miR-99a-5p* in LUAD cells.

| Genes | *p*-value | Annotations |
|---|---|---|
| *CKS1B* *CENPF* *CDC25A* *FAM64A* | $4.14 \times 10^{-5}$ | (GO) 0051301: cell division |
| *CKS1B* *CENPF* *CDC25A* *FAM64A* | 0.000137009 | (GO) 0007049: cell cycle |
| *CKS1B* *CDC25A* | 0.00127197 | (GO) 0000079: regulation of cyclin-dependent protein serine/threonine kinase activity |
| *CDC25A* | 0.00529465 | (GO) 0110032: positive regulation of G2/MI transition of meiotic cell cycle |
| *CKS1B* *CDC25A* | 0.00547439 | (GO) 0008283: cell population proliferation |

**Table S4B.** Significantly enriched annotations regulated by *miR-99a-3p* in LUAD cells.

| Genes | *p*-value | Annotations |
|---|---|---|
| *ZWINT*<br>*CDKN3*<br>*CDK1*<br>*NEK2*<br>*MKI67*<br>*MCM4*<br>*KIF11*<br>*ESCO2*<br>*SGOL1*<br>*NCAPG*<br>*CASC5*<br>*FANCI*<br>*FAM64A* | $9.15 \times 10^{-14}$ | (GO) 0007049: cell cycle |
| *ZWINT*<br>*CDK1*<br>*NEK2*<br>*KIF11*<br>*SGOL1*<br>*NCAPG*<br>*CASC5*<br>*FAM64A* | $1.10 \times 10^{-7}$ | (GO) 0051301: cell division |
| *NEK2*<br>*ESCO2*<br>*SGOL1*<br>*CASC5* | $7.25 \times 10^{-5}$ | (GO) 0007059: chromosome segregation |
| *ZWINT*<br>*NEK2*<br>*SGOL1* | 0.000165787 | (GO) 0000070: mitotic sister chromatid segregation |
| *NEK2*<br>*KIF11* | 0.000243324 | (GO) 0046602: regulation of mitotic centrosome separation |

**Table S4C.** Pathways regulated by *miR-99a-5p* in LUAD cells.

Cell division pathway

| Gene symbol | Gene name |
| --- | --- |
| *CKS1B* | CDC28 protein kinase regulatory subunit 1B |
| *CENPF* | Centromere protein F |
| *CDC25A* | Cell division cycle 25A |
| *FAM64A* | Family with sequence similarity 64 member A |

Cell cycle pathway

| Gene symbol | Gene name |
| --- | --- |
| *CKS1B* | CDC28 protein kinase regulatory subunit 1B |
| *CENPF* | Centromere protein F |
| *CDC25A* | Cell division cycle 25A |
| *FAM64A* | Family with sequence similarity 64 member A |

Regulation of cyclin-dependent protein serine/threonine kinase activity pathway

| Gene symbol | Gene name |
| --- | --- |
| *CKS1B* | CDC28 protein kinase regulatory subunit 1B |
| *CDC25A* | Cell division cycle 25A |

Positive regulation of G2/MI transition of meiotic cell cycle pathway

| Gene symbol | Gene name |
| --- | --- |
| *CDC25A* | Cell division cycle 25A |

Cell population proliferation pathway

| Gene symbol | Gene name |
| --- | --- |
| *CKS1B* | CDC28 protein kinase regulatory subunit 1B |
| *CDC25A* | Cell division cycle 25A |

**Table S4D.** Pathways regulated by *miR-99a-3p* in LUAD cells.

Cell cycle pathway

| Gene symbol | Gene name |
|---|---|
| *ZWINT* | ZW10 interacting kinetochore protein |
| *CDKN3* | Cyclin dependent kinase inhibitor 3 |
| *CDK1* | Cyclin dependent kinase 1 |
| *NEK2* | NIMA related kinase 2 |
| *MKI67* | Marker of proliferation Ki-67 |
| *MCM4* | Minichromosome maintenance complex component 4 |
| *KIF11* | Kinesin family member 11 |
| *ESCO2* | Establishment of sister chromatid cohesion N-acetyltransferase 2 |
| *SGOL1* | Shugoshin 1 |
| *NCAPG* | Non-SMC condensin I complex subunit G |
| *CASC5* | Cancer susceptibility candidate 5 |
| *FANCI* | FA complementation group I |
| *FAM64A* | Family with sequence similarity 64 member A |

Cell division pathway

| Gene symbol | Gene name |
|---|---|
| *ZWINT* | ZW10 interacting kinetochore protein |
| *CDK1* | Cyclin dependent kinase 1 |
| *NEK2* | NIMA related kinase 2 |
| *KIF11* | Kinesin family member 11 |
| *SGOL1* | Shugoshin 1 |
| *NCAPG* | Non-SMC condensin I complex subunit G |
| *CASC5* | Cancer susceptibility candidate 5 |
| *FAM64A* | Family with sequence similarity 64 member A |

Chromosome segregation pathway

| Gene symbol | Gene name |
|---|---|
| *NEK2* | NIMA related kinase 2 |
| *ESCO2* | Establishment of sister chromatid cohesion N-acetyltransferase 2 |
| *SGOL1* | Shugoshin 1 |
| *CASC5* | Cancer susceptibility candidate 5 |

Mitotic sister chromatid segregation pathway

| Gene symbol | Gene name |
|---|---|
| *ZWINT* | ZW10 interacting kinetochore protein |
| *NEK2* | NIMA related kinase 2 |
| *SGOL1* | Shugoshin 1 |

Regulation of mitotic centrosome separation pathway

| Gene symbol | Gene name |
|---|---|
| *NEK2* | NIMA related kinase 2 |
| *KIF11* | Kinesin family member 11 |
